# Concomitant use of sivelestat sodium hydrate and antithrombotic drugs worsens the treatment outcome of patients with acute respiratory distress syndrome and suppression of fibrinolysis: a single-center, retrospective study

**DOI:** 10.20407/fmj.2024-026

**Published:** 2025-04-17

**Authors:** Takahiro Kato, Tomohiro Mizuno, Takenao Koseki, Kazuo Takahashi, Shigeki Yamada, Kazuyoshi Imaizumi, Naotake Tsuboi, Naozumi Hashimoto

**Affiliations:** 1 Department of Pharmacotherapeutics and Informatics, Fujita Health University, School of Medicine, Toyoake, Aichi, Japan; 2 Department of Biomedical Molecular Sciences, Fujita Health University, School of Medicine, Toyoake, Aichi, Japan; 3 Department of Respiratory Medicine, Fujita Health University, School of Medicine, Toyoake, Aichi, Japan; 4 Department of Nephrology, Fujita Health University, School of Medicine, Toyoake, Aichi, Japan

**Keywords:** Acute respiratory distress syndrome, Sivelestat sodium hydrate, Fibrinolysis suppression, Antithrombotic drug

## Abstract

**Objectives:**

Sivelestat sodium hydrate (SSH) may be effective in the early stage of acute respiratory distress syndrome (ARDS) before the neutrophil extracellular trap scaffold structure is complete. Therefore, patients with suppression of fibrinolysis (SF) before the secondary fibrinolytic process might benefit from SSH administration. The primary aim of this study was to determine the effect of the SF state and combination therapy on the effect of SSH administration.

**Methods:**

We retrospectively reviewed the data of patients diagnosed with ARDS at Fujita Health University Hospital between July 2005 and December 2016. Patients with ARDS were stratified into the SF and hyperfibrinolysis (HF) groups. Using the fibrin degradation product (FDP)/D-dimer ratio, cut-off values were set as follows: FDP/D-dimer >2 for the HF group and FDP/D-dimer ≤2 for the SF group. The 28-day mortality was the primary endpoint.

**Results:**

In total, 168 patients (71 in the HF group and 97 in the SF group) were included in the analysis. The mortality within 28 days was not different based on SSH administration in either group (HF group: *p*=0.956, SF group: *p*=0.957). In the SF group, the mortality rate within 28 days in SSH-treated patients who received antithrombotic drugs was significantly higher than that in patients who received SSH only (*p*<0.05). However, this finding was not present in the HF group (*p*=0.786).

**Conclusions:**

Concomitant use of SSH and antithrombotic drugs might worsen the treatment outcome of patients with ADRS in the SF state.

## Introduction

Acute respiratory distress syndrome (ARDS) is an acute respiratory failure characterized by non-cardiogenic pulmonary edema secondary to various underlying conditions or trauma, such as sepsis or severe pneumonia. ARDS exhibits distinct characteristics in pathology such as diffuse alveolar damage with the accumulation of neutrophils in the lungs and alveolar spaces.^1^ A previous study reported that 10% of intensive care unit (ICU) admissions and 23% of patients on mechanical ventilation fulfilled the criteria for ARDS.^2^ Many patients with coronavirus disease 2019 are treated without mechanical ventilation. Therefore, the net incidence of ARDS may be higher than that reported previously.^3^ Furthermore, the in-hospital mortality rate remains high, with that of moderate-to-severe ARDS reaching 40% after 90 days.^4^ Unfortunately, clinical trials of novel ARDS pharmacotherapies have not demonstrated consistent efficacy.^1^

Neutrophil extracellular trap (NET) formation is observed in the small pulmonary vessels of patients who succumb to severe ARDS,^5^ and the release of NETs is the primary function of neutrophils.^6^ The levels of inflammatory cytokines, such as interleukin-6 and interleukin-8, which induce NETosis, are increased in patients with severe ARDS.^7,8^ NETs are extracellular decondensed chromatin molecules decorated with histones and antimicrobial proteins that physically trap and kill microbes. NETs contain several proteins, such as histones, the serine protease neutrophil elastase (NE), and myeloperoxidase.^9^ NE promotes endothelial injury and increases vascular permeability, leading to the pathogenesis of ARDS.^10,11^

Sivelestat sodium hydrate (SSH), which is a selective NE inhibitor developed in Japan, was first used in 2002 for treating ARDS. Although no significant difference in the 30-day mortality rate was observed in a domestic phase III trial using SSH, the duration of mechanical ventilation and length of stay in the ICU were reduced in patients with acute lung injury.^12^ This finding is supported by a phase IV study that reevaluated the efficacy and safety of SSH, and it showed early weaning from mechanical ventilation.^13^ In a retrospective study, SSH administration reduced the disseminated intravascular coagulation (DIC) scores in patients with ARDS and DIC.^14^ A meta-analysis also showed that SSH not only shortened the duration of ICU admission but also improved the oxygenation index in patients with ARDS.^15^ However, an international, multicenter, double-blind trial (STRIVE trial) showed an increase in the 180-day mortality rate in the SSH-treated group.^16^ The efficacy of SSH on outcomes in patients with ARDS is limited, and the ARDS Clinical Practice Guideline 2021 does not recommend the use of SSH in patients with ARDS.^17^

In the early stage of ARDS, lung endothelial damage triggers inflammation by activated neutrophils, resulting in hypercoagulation and vascular hyperpermeability. This process amplifies the crosstalk between NET production by activated neutrophils and fibrin deposition.^18^ NET formation is also efficiently stimulated by activated platelets.^19^ SSH suppresses reactive oxidative species-associated NET formation.^20^ Therefore, patients with hypercoagulation or suppression of fibrinolysis (SF) before the secondary fibrinolytic process might benefit from SSH administration. Furthermore, the efficacy of SSH may be more clearly evaluated by comparing the presence or absence of concomitant antithrombotic drugs that affect the coagulation–fibrinolytic system or platelet aggregation. Previous reports have suggested that SSH has partial efficacy in secondary endpoints, such as the ICU discharge rate, survival rate, and ventilator-free days.^12,13,15^ The optimization of treatment timing and combination therapy might improve the potential of SSH as a treatment option for ARDS.

Although clinical indicators for classifying SF and hyperfibrinolysis (HF) states have not been fully established, the fibrinogen and fibrin degradation product (FDP)/D-dimer ratio is used for classifying patients with DIC into the state of SF or HF.^21,22^ In this study, we used the FDP/D-dimer ratio to classify the coagulation–fibrinolytic state in patients with ARDS. We compared mortality between patients with HF or SF to evaluate the hypothesis that SSH is not effective in patients with HF. In addition, we assessed the effect of concomitant antithrombotic drugs on the effect of SSH in patients in the SF or HF state.

## Methods

### Study design

Patients who had acute onset, bilateral infiltrates as shown by an X-ray or computed tomography scan, no left atrial hypertension observed or respiratory failure not explained by heart failure or fluid overload, and a PaO_2_/FIO_2_ (P/F) ratio ≤300 were diagnosed with ARDS and were enrolled in this single-center, retrospective study. The study was conducted between July 2005 and December 2016 at Fujita Health University Hospital. The cut-off values for coagulation–fibrinolytic abnormalities were defined as follows: an FDP/D-dimer ratio >2 indicated a tendency for HF and an FDP/D-dimer ratio ≤2 indicated a tendency for SF.^21–24^ The observation period ranged from 1 week before the date of diagnosing ARDS by the American-European Consensus Conference definition^25^ or the Berlin definition^26^ to 28 days after SSH administration. Patients <18 years of age and those without FDP, D-dimer, and P/F ratio measurements were excluded. All procedures in this study were performed in accordance with the ethical standards of our institution and the principles of the Declaration of Helsinki of 1964. This study was approved by the Ethics Committee of the Fujita Health University School of Medicine (Approval Code: HM-23-102, date of approval: 3 July 2023). An opt-out approach approved by the ethics board was used to obtain informed consent.

### Data acquisition

Baseline data, such as age, sex, the systemic inflammatory response syndrome (SIRS) score, the date of diagnosing ARDS, the date of death, and discharge, were obtained from electronic medical records. The SIRS score was assessed on the day that ARDS was diagnosed and was scored according to the American College of Chest Physicians/Society of Critical Care Medicine Consensus Conference.^27^ Laboratory test results, such as the prothrombin time-international normalized ratio, platelet count, FDP and D-dimer ratio, and P/F ratio based on an arterial blood gas analysis, were obtained from electronic medical records within 1 week before the date of diagnosing ARDS. The data closest to the date of diagnosis were used if the analysis was performed more than once. Information on comorbidities, the etiology of ARDS, the infection site, the day of SSH administration after diagnosis of ARDS, and concomitant medications was also obtained from the electronic medical records.

We investigated the etiology of ARDS according to the risk factors described in the Berlin definition.^26^ Concomitant medications, which were antithrombotic drugs (aspirin, warfarin, heparin, clopidogrel, cilostazol, and dipyridamole) and glucocorticoids, were defined as those administered within 1 week before the diagnosis of ARDS. Sepsis, solid tumors, and abdominal aortic aneurysms were investigated as typical diseases that affect the coagulation–fibrinolysis system.^28^

### Outcome measures

In the SF and HF groups, the efficacy of SSH was evaluated using the mortality rate within 28 days as the primary endpoint. To assess the effect of antithrombotic drugs on the effect of SSH, we compared mortality within 28 days with and without concomitant antithrombotic drugs in patients who were treated with SSH.

### Statistical analysis

Values indicating a non-normal distribution are presented as the median and interquartile range. The Mann–Whitney *U*-test was used to compare the median of continuous variables, and the χ^2^-test was used to compare the proportion of categorical variables. The Kaplan–Meier curve with the log-rank test was used to assess the relationship between mortality within 28 days. A *p* value of <0.05 was considered to indicate a statistically significant difference. All statistical analyses were performed using the Statistical Package for the Social Sciences for Windows (version 24.0; IBM Corp., Armonk, NY, USA).

## Results

### Patients’ data

**Figure 1:**
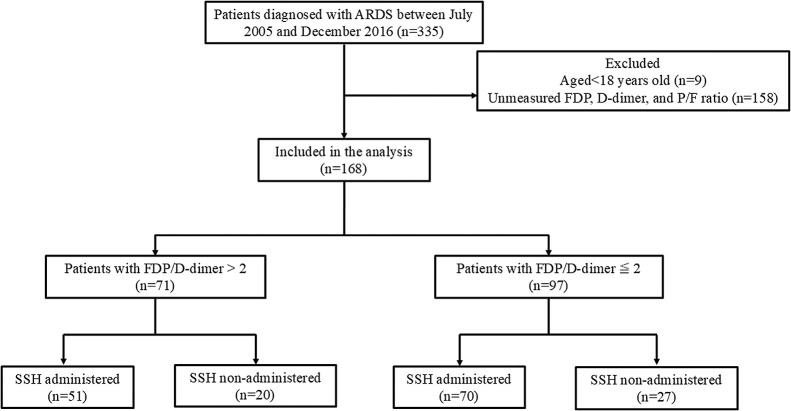
Flow chart of the study groups. Abbreviations: ARDS, acute respiratory distress syndrome; FDP, fibrin degradation product; SSH, sivelestat sodium hydrate.

In this study, we included 168 patients with ARDS, who were subsequently divided into the HF (n=71) and SF groups (n=97) on the basis of cut-off values for coagulation–fibrinolytic abnormalities. The number of patients who received SSH was 51 (71.8%) in the HF group and 70 (72.2%) in the SF group (Figure 1). The baseline characteristics of the patients with ARDS are shown in Table 1. In the HF and SF groups, there were no significant differences in the patients’ age, proportion of male sex, prothrombin time, platelet count, P/F ratio, or proportion of infection sites between patients with or without SSH administration. The day of SSH administration after the diagnosis of ARDS was the same in the two groups. SIRS scores were significantly higher in patients treated with SSH than in those who were not treated with SSH in the SF group (*p*=0.017). In the SF group, the proportion of patients who received antithrombotic drugs was significantly higher in those with SSH administration than in those without SSH administration (*p*=0.013). However, there was no significant difference in the number of patients who received glucocorticoid therapy.

### Comparison of mortality within 28 days

**Figure 2:**
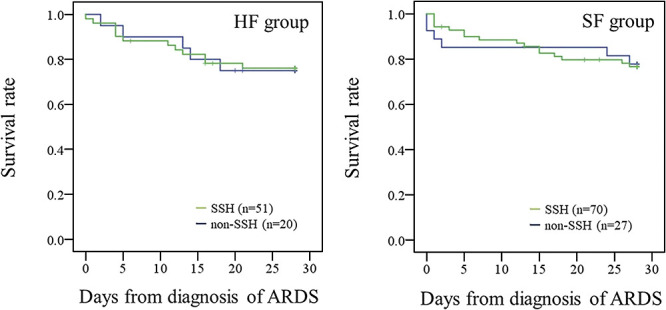
Survival rate from the diagnosis of ARDS. The survival rates were 76.5% (SSH) and 75.0% (non-SSH) in the HF group. The survival rates were 77.1% (SSH) and 77.8% (non-SSH) in the SF group. Abbreviations: ARDS, acute respiratory distress syndrome; SSH, sivelestat sodium hydrate.

**Figure 3:**
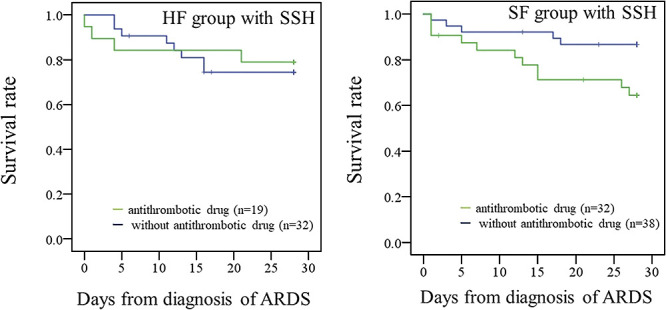
Survival rate within 28 days in the HF and SF groups with SSH administration. The survival rates were 78.9% (antithrombotic drugs) and 75.0% (without antithrombotic drugs) in the HF group. The survival rates were 65.6% (antithrombotic drugs) and 86.8% (without antithrombotic drugs) in the SF group. *p<0.05 vs without antithrombotic drugs (log-rank test). Abbreviations: ARDS, acute respiratory distress syndrome; SSH, sivelestat sodium hydrate; SIRS, systemic inflammatory response syndrome.

The primary endpoint is shown in Figures 2 and 3. The mortality rates were 23.5% (SSH) and 25.0% (non-SSH) in the HF group. The mortality rates were 22.9% (SSH) and 22.2% (non-SSH) in the SF group. The mortality rate within 28 days was not different based on SSH administration in either group (HF group: *p*=0.956, SF group: *p*=0.957) (Figure 2). The mortality rates within 28 days in the HF and SF groups with SSH administration are shown in Figure 3. We compared the mortality rate between patients with or without concomitant use of antithrombotic drugs. In the SF group, the log-rank-test showed that mortality within 28 days in SSH-treated patients who received antithrombotic drugs was significantly higher than that in patients who received SSH only (*p*<0.05). However, this finding was not present in the HF group (*p*=0.786). The mortality rates were 21.1% (antithrombotic drugs) and 25.0% (without antithrombotic drugs) in the HF group. The mortality rates were 34.4% (antithrombotic drugs) and 13.2% (without antithrombotic drugs) in the SF group.

## Discussion

We hypothesized that SSH and concomitant antithrombotic drugs affect mortality in patients with ARDS in the HF or SF state. Therefore, we investigated the efficacy of SSH in patients diagnosed with ARDS who were classified according to the FDP/D-dimer cut-off value.

### Effect of SSH on survival

The primary endpoint of the present study was mortality. Therefore, the severity of ARDS in patients who received SSH needed to be similar to compare the HF or SF state in relation to mortality. There was no significant difference in the P/F ratio between the HF and SF groups treated with and without SSH, and no clinical bias in the severity of ARDS was observed. SSH administration did not improve the 28-day mortality rate in the SF group, and no effect of SSH was observed, regardless of the SF or HF state. SSH decreased the mortality rate in ≤5/1,000 patients in four randomized, controlled trials. Therefore, the ARDS Practice Guidelines 2021 recommend not using SSH in patients with ARDS because these effects were judged as small.^17^ Our results support the recommendation of this guideline.

### Combination therapy with SSH and antithrombotic drugs

In the SF group, the proportion of patients who received antithrombotic drugs was higher in those who received SSH than in those who did not. Generally, when NETs are suddenly released because of external stimuli, such as bacteria or chemical mediators, excessive inflammation, coagulation, and platelet aggregation reactions occur, which are detrimental to the organism. Therefore, antithrombotic activity and systemic tissue damage might be suppressed by the administration of antithrombotic drugs. However, antithrombotic drugs increased the 28-day mortality rate in patients who received SSH in the SF group in our study. Regarding the relationship between the components released from NETs and thrombus formation, extracellular histones promote intrinsic and extrinsic blood coagulation pathways.^29^ In addition, tissue factors and other factors bind to the NET surface and contribute to coagulation activation and platelet thrombus formation.^30^ NET production might enhance the activation of intrinsic and extrinsic blood coagulation pathways in the SF state more than in the HF state. Thrombus formation by NET release plays a role in the innate immune mechanism that prevents microorganisms from spreading systemically.^31^ Antithrombotic drugs may inhibit thrombus formation by NETs, reducing the risk of organ ischemia and suppressing systemic tissue damage. However, our results suggest that inhibition of thrombogenicity by the combination of antithrombotic therapy and SSH progressively weakens the local control of infection from the perspective of innate immune mechanisms in patients in the SF state. This possibility suggests the importance of ensuring a balance between maintaining organ perfusion and controlling the spread of infection before deciding whether to add SSH to patients with SF state antithrombotic drugs. To the best of our knowledge, this is the first report to focus on a coagulation–fibrinolytic system in patients with ARDS who were treated with SSH. Therefore, the results of our study suggest the importance of deciding on the use of SSH and antithrombotic drugs on the basis of activity of the coagulation–fibrinolytic system when evaluating the efficacy of SSH.

Our study has some limitations. First, this was a retrospective, single-center study with a small sample size. However, performing a prospective study is not practical because the ARDS Clinical Practice Guideline 2021 does not currently recommend the use of SSH.^17^ Second, the diagnostic criteria for ARDS were revised from the old definition (American-European Consensus Conference definition) to the new definition (Berlin definition) after 2012.^32^ Therefore, the two definitions were combined in the present study, which differs from the current ARDS treatment. Third, in addition to SSH, patients received adjuvant treatments, such as antibiotic treatment for infection, fluid therapy, and ventilator support. Additionally, the effects of the treatment duration, dosage, and ventilator setting conditions could not be investigated because of a lack of information. Fourth, there were a certain number of patients with no obvious etiology, and thus the cause of ARDS was unknown. An international, prospective cohort study of a large group of patients with ARDS showed that the etiology of ARDS in 8.3% of all patients was unexplained, indicating difficulty in diagnosing the cause of ARDS.^2^ This lack of an obvious etiology is one of the factors that influenced our results. Therefore, an additional investigation of the etiology is important in the future. Fifth, although anticoagulants and antiplatelet drugs have different mechanisms of action, previous studies have shown that aspirin, unfractionated heparin, and low molecular weight heparin decrease the release of NETs.^33^ However, the mechanism of how these drugs are involved in the effect of SSH is unclear. Therefore, further studies on this issue are required. Sixth, because the attending physician decided whether to administer SSH, treatment outcomes may have been affected by factors other than the SSH treatment efficacy. In addition, selection bias may have caused alpha errors in this study because patients with a worse clinical course might have had a greater chance of being administered antithrombotic drugs.

In conclusion, the concomitant use of SSH and antithrombotic drugs might worsen the treatment outcome of patients with ARDS in the SF state.

## Figures and Tables

**Table 1:** Characteristics of patients with acute respiratory distress syndrome

Characteristic	HF group (n=71)			SF group (n=97)		
	SSH (n=51)	Non-SSH (n=20)	*p* value	SSH (n=70)	Non-SSH (n=27)	*p* value
Age (years), median (IQR)	71.0 (58.0–79.0)	71.0 (58.0–78.5)	0.774^a^	69.0 (54.0–77.0)	69.5 (54.8–77.0)	0.308^a^
Male sex, n (%)	36 (70.6)	15 (75.0)	0.710^b^	53 (75.7)	19 (70.4)	0.590^b^
SIRS score, median (IQR)	2.0 (1.0–3.0)	2.0 (1.0–3.0)	0.253^a^	2.0 (1.0–3.0)	2.0 (1.0–3.0)	0.017^a^
SIRS score (≥3), n (%)	22 (43.1)	4 (20.0)	0.069^b^	36 (51.4)	6 (22.2)	0.009^b^
PT-INR, median (IQR)	1.12 (1.04–1.36)	1.13 (1.05–1.38)	0.990^a^	1.21 (1.11–1.33) (n=67)	1.22 (1.12–1.34)	0.517^a^
PLT (×10^9^/L), median (IQR)	16.5 (9.9–22.7)	16.5 (9.9–22.7)	0.618^a^	14.8 (7.9–22.9) (n=63)	15.5 (8.0–23.4)	0.223^a^
PaO_2_/FIO_2_ ratio, median (IQR)	150.0 (106.1–231.0)	152.1 (106.1–238.6)	0.069^a^	151.3 (96.5–228.4)	150.3 (97.5–229.8)	0.885^a^
Etiology of ARDS, n*						
Acute pneumonia	20	7	N/A	21	6	N/A
Aspiration	9	1	N/A	12	2	N/A
Pulmonary contusion	1	1	N/A	12	0	N/A
Sepsis	24	5	N/A	33	16	N/A
Acute pancreatitis	0	0	N/A	1	0	N/A
Trauma	1	2	N/A	12	2	N/A
Burn injury	0	1	N/A	4	0	N/A
Other	8	5	N/A	7	1	N/A
Unknown	11	7		14	3	
Concomitant diseases, n*						
Hypertension	17	6	N/A	11	5	N/A
Diabetes	15	3	N/A	10	5	N/A
Ischemic heart disease	10	1	N/A	8	4	N/A
Infection site, n*						
Chest	36	12	N/A	41	19	N/A
Abdomen	2	0	N/A	10	2	N/A
Blood stream	13	4	N/A	16	8	N/A
Other	11	6	N/A	19	7	N/A
The day of SSH administration after diagnosing ARDS, median (IQR)	6.5 (4.0–12.8)	N/A		7.0 (4.0–13.0)	N/A	0.057^a^
Concomitant medications, n (%)						
Antithrombotic drugs	19 (37.3)	5 (25.0)	0.326^b^	32 (45.7)	5 (18.5)	0.013^b^
Aspirin*	5	1	N/A	9	2	N/A
Warfarin*	3	2	N/A	4	0	N/A
Heparin*	8	3	N/A	16	3	N/A
Clopidogrel*	2	0	N/A	2	0	N/A
Cilostazol*	1	0	N/A	5	0	N/A
Dipyridamole*	0	0	N/A	1	0	N/A
GC	15 (29.4)	4 (20.0)	0.420^b^	15 (21.4)	8 (29.6)	0.395^b^
Typical diseases affecting the coagulation–fibrinolysis system, n *						
Sepsis	29			49		N/A
Solid tumors	12			11		N/A
AAA	1			1		N/A

Data are presented as n (%) or median (IQR). Abbreviations: SIRS, systemic inflammatory response syndrome; PT-INR, prothrombin time-international normalized ratio; PLT, platelet count; FDP, fibrin/fibrinogen degradation product; GC, glucocorticoid; DD, D-dimer; SF, suppression of fibrinolysis; HF, hyperfibrinolysis; SSH, sivelestat sodium hydrate; AAA, abdominal aortic aneurysm. *p* values were obtained using the ^a^Mann–Whitney *U*-test or ^b^Chi-square test. *Includes duplicates.
